# The Successful Relaunch of Scientia Pharmaceutica Continues Achieving Success

**DOI:** 10.3797/scipharm.ed-11-02

**Published:** 2011-08-08

**Authors:** Wolfgang Kubelka, Gernot A. Eller

**Affiliations:** 1 Department of Pharmacognosy, University of Vienna, Vienna, Austria; 2 Department of Drug and Natural Product Synthesis, University of Vienna, Vienna, Austria

Since our relaunch in early 2008 [1], our journal experienced many changes and has developed very, very positively. In this short summary we will give you selected snapshots of what has happened, what is going on, and what is yet to come.

## Online version (‘open access’) and editorial work

While we completely modernized the print version with volume 79 (2011), and while it will continue to meet the needs of some of our readers, University libraries, scientific databases and indexing companies, the main focus lies on our online version on www.scipharm.at. Due to the generous support of our parent organizations, the Austrian Chamber of Pharmacists (ÖAK, Österreichische Apothekerkammer) and the Austrian Pharmaceutical Society (ÖPhG, Österreichische Pharmazeutische Gesellschaft), the electronic version of our journal can still be accessed on our homepage without any subscription or registration barriers. Such an open-access model has become more and more frequent in scientific publishing during the last decade. However, it is quite unusual neither readers (‘open access’) *nor* authors (no publishing fees) are charged, and this holds true for Scientia Pharmaceutica. This fact guarantees the editorial independence of Sci Pharm.

**Fig. 1:**
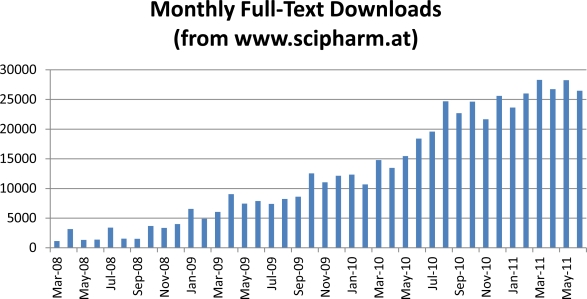
Full-text downloads (PDF) of articles published on www.scipharm.at (abstract-only-views are not included)

The popularity of our online version is still increasing. Figure 1, which shows the monthly full text downloads from our homepage, documents this. Please note, these incredible numbers do not include ‘abstract only views’ or the many views on internet sites that have hosted the content of our journal. Of course, these positive circumstances, which ensure that each paper published receives the best possible dissemination and publicity, attract many researchers from around the world as potential authors. Consequently, this led—and still leads—to an increased number of submissions (approximately 400 manuscripts are expected for 2011!).

**Fig. 2:**
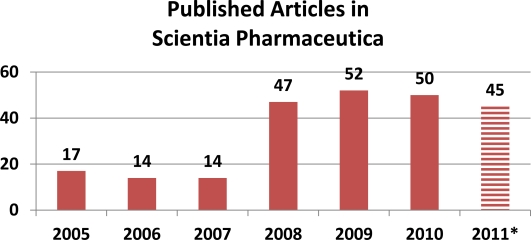
Number of published reviews, research papers, and short communications. * based on 3 of 4 issues.

**Fig. 3:**
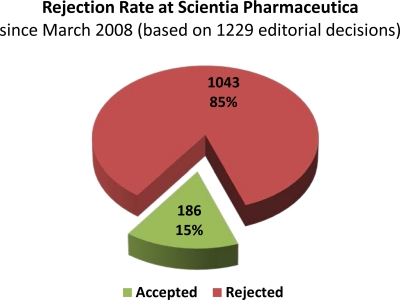
Rejection Rate

However, because of the expansion of our editorial team—Franz GABOR and Reinhard LÄNGER joined as scientific editors and many new members joined our scientific advisory board (www.scipharm.at/board/)—we are able administrate the rapid peer review process for all submissions. Nevertheless, because ‘quality comes first’ at Sci Pharm, only the best of the numerous submissions are selected by our expert editors for publication, with the highly appreciated help of many referees. Although we do not have a specific page or paper limit for publication, we are currently publishing around 50–60 papers each year (see figure 2). While comparing the number of submissions with the number of published articles, it is evident Sci Pharm applies a rigorous peer review for each paper to maintain the high scientific standards (see the current rejection rate of approximately 85% in figure 3).

## Special Issue: Novel Biogene Sesquiterpenes (NBST)

Under the guidance of five internationally renowned experts (Prof. Dr. Yoshinori Asakawa, Tokushima, Japan; Prof. Dr. Franz Bucar, Graz, Austria; Prof. Dr. Biswanath Das, Hyderabad, India; Prof. Dr. De-An Guo, Shanghai, People’s Republic of China; Prof. Dr. Andrew Marston, Bloemfontein, Republic of South Africa) a special issue on sesquiterpenes has been scheduled for 2012. The editors are looking forward to receiving your high-quality contributions. Further information including the official call-for-papers can be found online >>> www.scipharm.at/nbst/

## Indexing & Impact factor

Many indexing services have noticed the positive transformation of our journal which has resulted in an increased abstracting of the articles. Through the implementation of the digital object identifier (DOI-CrossRef) for each published article, a crosslinking from many of those databases to the full text on the Sci Pharm webpage is also possible. For example, the Chemical Abstracts Service reclassified Sci Pharm as Core journal, and EBSCO, CAB Abstracts, ProQuest, and many others have incorporated Sci Pharm into their products. Sci Pharm achieved one of its principal goals through its inclusion to PubMed, the well-known second-to-none database for biomedical literature. Also, all articles are placed into PubMed’s open-access archive PubMed Central.

**Fig. 4:**
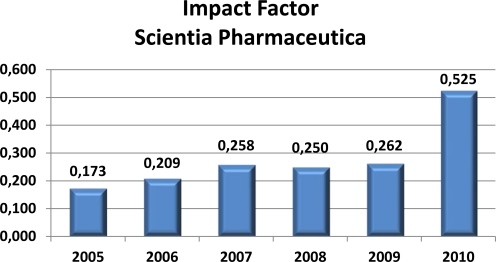
Unofficial impact factors for Scientia Pharmaceutica

Currently, the journal is applying for inclusion in Thomson Reuter’s database ‘Journal Citation Reports^®^’, which is better known for its associated journal metric the Impact Factor (IF)—a measure reflecting the average number of citations to articles published in a specific journal. You can help accelerate this process by recommending Sci Pharm on Thomson Reuter’s webpage http://science.thomsonreuters.com/info/journalrec/. However, even for journals that have not been tracked for enough time to have an Impact Factor or are not yet tracked by Thomson Reuters (ISI), it is possible to calculate an unofficial Impact Factor based on Thomson Reuter’s ‘Web of Science’ database, which is shown in figure 4.

Please note that the journal’s relaunch in 2008 is not evident before 2010 in this chart (because of the calculation algorithm of the IF based on the past two years). Indeed, the IF 2010 doubled compared to the nearly constant IF in the previous years. It is noteworthy that for 2011 a significantly further increased IF of about 0.7–0.9 is expected.

## Citation Award

This summer for the first time, Sci Pharm will grant an award to the author(s) of the most cited paper published in the journal. According to Thomson Reuter’s ‘Web of Knowledge’ database, all articles of 2009 were analyzed and the article with the most citations in 2010 qualified for this prize [2]:

*Biotransformation of Artemisinin Mediated through Fungal Strains for Obtaining Derivatives with Novel Activities*, by S. Srivastava, S. Luqman, A. Fatima, M. P. Darokar, A. S Negi, J. K. Kumar, K. Shanker, C. S. Chanotiya, S. Tandon, S. P. S. Khanuja, which was published in Sci Pharm, Volume 77, Issue 1 (2009), Pages 87–95 [3].

Sci Pharm plans to repeat this citation award in future years.

## Figures and Tables

**Fig. 5:**
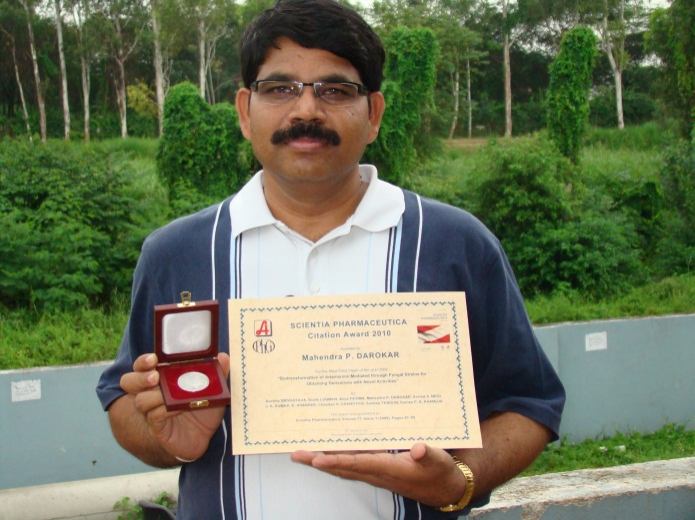
This year’s recipient, M. P. Darokar from Lucknow, India, received his ‘Citation Award’ certificate together with the 1 ounce Vienna Philharmonic Coin. This renowned Austrian coin shows the world famous Vienna Philharmonic Orchestra. It was coined in 2009, the year when the article was published. Congratulations!
